# Prevalence of antenatal depression and associated factors among pregnant women in Aneded woreda, North West Ethiopia: a community based cross-sectional study

**DOI:** 10.1186/s13104-019-4717-y

**Published:** 2019-10-30

**Authors:** Abebe Habtamu Belete, Mulunesh Alemayehu Assega, Amanuel Alemu Abajobir, Yihalem Abebe Belay, Mengistie Kassahun Tariku

**Affiliations:** 1 Department of Public Health, College of Health Sciences, Debre Markos University, Debre Markos, Ethiopia; 2 Department of Public Health, College of Medicine and Health Sciences, Bahir Dar University, Bahir Dar, Ethiopia; 3 Maternal and Child Wellbeing Unit, African Population and Health Research Centre, Nairobi, Kenya

**Keywords:** Prevalence, Antenatal depression, Pregnant women, Ethiopia

## Abstract

**Objectives:**

To assess the prevalence of antenatal depression and factors associated with antenatal depression among pregnant women in Aneded woreda, Northwest Ethiopia, 2019. A community based- cross sectional study was conducted in Aneded woreda among 7 kebles’ of North-West, Ethiopia from March 16 to April 23, 2019. A total of 342 pregnant women were recruited using simple random sampling.

**Result:**

The prevalence of antenatal depression was 15.20%. Urban residence [AOR = 6.8; 95% CI (1.97, 23.32)], marital status of being unmarried [AOR = 5.1; 95% CI (1.79, 14.63)], occupation of being government employee [AOR = 8.8; 95% CI (2.06, 37.12)] and merchant [AOR = 3.7; 95% CI (1.27, 10.91)], prim gravid [AOR = 5.3; 95% CI (2.03, 13.82)], not attend ANC follow up [AOR = 8.7; 95% CI (3.46, 21.79)], intimate partner violence [AOR = 4.5; 95% CI (1.28, 15.52)], unplanned pregnancy [AOR = 6.2; 95% CI (2.37, 16.06)], and substance use [AOR = 5.6; 95% CI (2.12, 14.92)] were significantly factors. Strengthen the risk prevention activities so important to tackle the problem of antenatal depression.

## Introduction

Antenatal depression is a type of depression that can be occurred during pregnancy [1]. The World Health Organization (WHO) estimates that by the year 2020, depression will be the second greatest cause of disease burden in both sexes and in pregnant women takes the lion’s share [2].

Currently, depression is considered to be a serious public health concern in the developing world that hinders maternal health improvement and is predicted to become the most common cause of disability [3].

Globally, 10% of pregnant women experience antenatal depression. In developing countries, antenatal depression is higher; it reaches up to 15.6% [4]. The prevalence of antenatal depression varies within sub-Sahara African countries from 47% in rural South Africa to 9.9% in Ghana. Pregnant women living in low and middle income countries are known to be highly risk for antenatal depression [5, 6].

In Ethiopia, depression is the top ten most leading cause of diseases burden [7] and antenatal depression affects as many as one in three pregnant women; almost half of those affected by depression have thoughts of ending their life [4, 8].

Antenatal depression is associated with adverse obstetric and fetal outcomes, including low birth weight [9], intrauterine growth restriction of fetus and preterm deliveries [10]. Antenatal depression contribute to maternal morbidity and mortality, in terms of poor health, increased disability and prolonged labor [10, 11].

The commonly identified risk factors of antenatal depression include mother’s age, education, employment status, marital status, substance use, pregnancy planned or not, parity and gravidity. Also intimate partner violence, gestational age, social support, low-income, and a history of antenatal care follow up were found to be the risk factors for antenatal depression [9, 10, 12–14].

The government of Ethiopia implements a mental health strategy of 2012/2013–2015/2016 which aimed to provide mental health services at all levels of the existing health system. The latest Health Sector Development Program (HSDP-IV) has set a target for integration of mental health into 50% of the healthcare settings by the end of the HSDP-IV in 2015 [12, 15].

Despite high concern of the government on maternal mental health, antenatal depression is a neglected issue in healthcare systems and remains a low priority in Ethiopian societies by different reasons. Previous studies had been focused on institutional based settings among antenatal care follow up pregnant women, but it is important to study the prevalence of antenatal depression and associated factors in population-based settings. Therefore, this research tried to assess the prevalence and factors associated with antenatal depression among pregnant women in Aneded woreda, Northwest Ethiopia.

## Main text

### Methods

#### Study setting and period

A community based cross-sectional study was conducted from March 16 to April 23, 2019 at Aneded woreda in Amhara Region, Ethiopia. It is located 18 km far away from Debre Markos town, 278 km from Addis Ababa, the capital city of Ethiopia and 260 km away from Bahir Dar the town of Amhara regional state.

The woreda has total populations of 110,326 total, 27,549 reproductive age groups and 3526 pregnant women. It has 19 rural and three urban kebeles, and there are 20 health posts and four health centers in the woreda [16].

#### Sample size and sampling techniques

Single population proportion was used to estimate the required sample size. It was calculated by assumption of 11.8% prevalence of antenatal depression in Debre Tabor [17], 95% of confidence interval, and 10% non-response rate. The total sample size was 162. Then adding design effect of two to reduce the variability it becomes 356.

Therefore, the final sample size was 356. The sample size was proportionally allocated for seven kebeles based on the number of pregnant women. Households with registered pregnant women in community health information system were selected by simple random sampling technique.

#### Measurement

The data collection tool comprised a structured face to face interviewer administered pretested questionnaire and the translated Amharic version of the Beck Depression Inventory (BDI-II) scale was used to detect depression. It is a reliable and valid measure of depression used and has been validated for use during pregnancy in different countries and settings including Ethiopia [10, 18, 19]. It consists of 21 items; each item describes a specific symptom of depression. Each statement scored on a 4-point scale (0 to 3) and total score obtained by summing the ratings for each statement. Therefore, the total score ranges from 0 to 63 [20–22]. Pregnant women with a score of less than 16 were considered as normal while women with BDI score of 16 and above considered as depressed [19]. All factors related questions were used to assess characteristics of study participants during pregnancy whereas substance use related questions were used to assess ever use of substance.

Training was given for data collectors and supervisors. Regular supervision and immediate feedback were carried out daily by the principal investigator on the data collection period.

#### Statistical analysis

Collected data were coded and entered by epi data version 3.1, and then it was exported to STATA version 14.1 for analysis. Descriptive statistics were used to describe categorical variables. Bi-variable analyses were done and all variables were entered into the multivariable regression model. Independent variables with a P-value < 0.05 with 95% confidence interval identified as statistically significant factors.

### Results

The median age of pregnant women was 24 years with interquartile range of 18 to 28 years.

#### Prevalence and factors associated with antenatal depression

In this study, the prevalence of antenatal depression was 15.20% (Table 1).

**Table 1:** Characteristics of pregnant women related to in Aneded woreda, Northwest Ethiopia, 2019 (N = 342)

Characteristics	Category	Frequency	Percent
Age	15–19	108	31.58
	20–29	184	53.80
	≥ 30	50	14.62
Occupation	Housewife	248	72.51
	Government employee	28	8.19
	Merchant	66	19.30
Residence	Urban	27	7.89
	Rural	315	92.11
Marital status	Unmarried**	31	9.06
	Married	311	90.94
Wealth index	Poorest	76	22.2
	Poor	97	28.36
	Middle	57	16.67
	Richer	86	25.15
	Richest	26	7.60
Gravidity	Prim gravid	83	24.27
	Multi gravid	259	75.73
Pregnancy planed	Yes	264	77.19
	No	78	22.81
ANC follow up	Yes	259	75.73
	No	83	24.27
Intimate partner violence	Yes	24	7.02
	No	318	92.98
substance use	Yes	53	15.49
	No	289	84.5

*Statisically significance (P < 0.05)

**Single, divorced and widowed

The study revealed that occupation of pregnant women had a significant association with antenatal depression in which the odd of depression was 8.8 times higher among government employees [AOR = 8.8; 95% CI (2.06, 37.12)] and 3.7 times higher among merchants [AOR = 3.7; 95% CI (1.27, 10.91)] as compared to housewives. Pregnant women who had lived in urban areas were 6.8 times [AOR = 6.8, 95% CI (1.97, 23.32)] more likely depressed as compared to pregnant women who had lived in rural areas (Table 2).

**Table 2:** Bi-variable and multivariable analyses result of antenatal depression among pregnant women in Aneded woreda, 2019

Variables	Antenatal depression		COR (95% CI)	AOR (95% CI)	P-value
	Depressed	Normal			
Age in year					
15–19	25	83	1	1	0.06
20–29	24	160	0.5 (0.25, 0.93)	0.6 (0.21, 1.44)	0.22
≥ 30	3	47	0.2 (0.06, 0.74)	0.2 (0.02, 1.25)	0.08
Occupation status					
House wife	28	220	1		
Government employee	11	17	5.1 (2.2, 11.9)	8.8 (2.06, 34.40)*	0.001
Merchant	13	53	1.9 (0.94, 3.97)	3.7 (1.27, 10.91)*	0.01
Residence					
Urban	12	15	5.5 (2.4, 12.6)	6.8 (1.97, 23.32)*	0.001
Rural	40	275	1		
Marital status					
Unmarried**	14	17	5.9 (2.7, 12.97)	5.1 (1.79, 14.63)*	0.001
Married	38	273	1		
Wealth index					
Poorest	5	71	0.2 (0.10, 0.62)	0.1 (0.07, 1.23)	0.12
Poor	9	88	0.3 (0.13, 0.52)	0.2 (0.14, 1.57)	0.09
Middle	3	54	0.1 (0.04, 0.63)	0.09 (0.05, 1.08)	0.07
Rich	35	77	1	1	0.15
Gravidity					
Yes	20	239	1		
No	32	51	7.5 (3.97, 14.2)	5.3 (2.03, 13.82)*	0.001
Pregnancy planning					
Yes	28	236	1		
No	24	54	3.75 (2.02, 6.97)	6.2 (2.37, 16.06)*	0.001
ANC follow up					
Yes	21	238	1		
No	31	52	6.76 (3.6, 12.7)	8.7 (3.46, 21.79)*	0.001
IPV					
Yes	13	11	8.46 (3.54, 20.2)	4.5 (1.28, 15.52)*	0.01
No	39	279	1		
Substance use					
Yes	25	28	8.7 (4.44, 16.92)	5.6 (2.12, 14.92)*	0.001
No	27	262	1		

*Statisically significance (P < 0.05)

**Single, divorced and widowed

Prim gravid women were 5.3 times [AOR = 5.3, 95% CI (2.03, 13.82)] more likely to be depressed as compared to women with who had history of previous pregnancy. Pregnant women who had not planned their current pregnancy were 6.2 times [AOR = 6.2, 95% CI (2.37, 16.06)] more likely to have antenatal depression than those women of who planned their current pregnancy (Table 2).

Women who had not ANC follow up were 8.7 times [AOR = 8.7, 95% CI (3.46, 21.79)] at higher odds to develop depression as compared to with those pregnant women who have had antenatal care follow up (Table 2).

The odds ratio revealed that those pregnant women who have suffered intimate partner violence were 4.5 times [AOR = 4.5, 95% CI (1.28, 15.52)] more likely to experience depression during their pregnancy than those pregnant women of without this history (Table 2).

### Discussion

The prevalence of antenatal depression was 15.20% [(95% CI (11.57, 19.46)].

The finding was consistent with a study done in Debre Tabor town (11.8%) [17], in Dubti Hospital Northeast Ethiopia (17.9%) [10], in South Asians (17.5%) [23], at Khartoum maternity hospital in Sudan (13.4%) [24], and in State of South Minas Gerais, Brazil (14.8%) [25] and lower than the findings from Gondar university hospital, Northwest Ethiopia (23%) [18], Maichew town, North Ethiopia (31.1%) [6], Adama (31.2%) [19], Shashemane (25.6%) [26], Addis Ababa, Ethiopia (24.94%) [27], Mombasa, Kenya (20%) [28], Nairobi, Kenya (32.9) [8], Nigeria (24.5%) [29], Western Saudi Arabia (57.5%) [25], India (36.75%) [30], Pakistan (18–80%) [31], Karachi, Pakistan (81%) [32] and Middle Easterners (19.5%) [33].

In other way, the current finding was higher than study done in Western Europeans (8.6%) [33].

The difference might be due to socio demographic, socio economic and cultural variations.

Moreover, this study also identified factors that have association with antenatal depression and found that residence in urban, occupation of being government employee and merchant, marital status of being unmarried, prim gravidity, unplanned pregnancy, not attend antenatal care follow up, intimate partner violence, and psychoactive substance use were factors that had significantly associated with antenatal depression.

Being government employee and merchant in this study, compared to being house wife was associated with depression, which increases the odds of endorsing depression during pregnancy by nine and four times respectively. This is different from the study done in Gondar university hospital, Northwest Ethiopia [18]. This difference could be pregnant women of government employee in this study area engage in triple burden duties as they are working to support their families with work outside their homes, at the same time carrying full responsibility for childcare and government employer. And also, those government employers are paid the minimum Ethiopian monthly wage which leads to unable cope up with the current economic inflation including the baby dipper cost, milk, house rent, social payment and consumable foods. Pregnant women of merchant due to fear of decreasing their income after delivery lead to difficulties in managing their household food security.

The prevalence of antenatal depression was seven times higher among women living in urban areas than those living in rural areas. The possible explanation for this finding might be the risk factors for antenatal depression that were unequally distributed across geographic areas and currently in urban areas social capital is loose. Fear of downhill and mostly economic inflation are the major responsible conditional situations that lead depression in urban area.

Unmarried women were five times more likely to have antenatal depression as compared to those women who had married. Similar finding was reported from other studies [6, 26]. This might be explained that no social support, poor financial capacity, culture of unmarried mother and psychological issues may in turn contribute to antenatal depression.

Prim gravid women were five times more likely to be depressed as compared to those of multi gravid. This finding is consistent with a study done in Gondar university hospital, Northwest Ethiopia [18]. This might be prim gravid women have not previous pregnancy experience; fear of childbirth and responsibility of child care is more common among primigravid which could aggravate the occurrence of depression.

Pregnant women who did not plan their current pregnancy were six times more likely to develop antenatal depression as compared with pregnant women who planned their current pregnancy. This finding is consistent with a study done in Addis Ababa [27], in Debre Tabor town, Northwest Ethiopia [17]. This might be due to women who did not plan their current pregnancy were getting into additional family burden, may have another imminent plan and it may be source of conflict in the household that leads to antenatal depression.

Pregnant women who were not attending ANC follow up were nine times more likely to develop antenatal depression as compared to pregnant women who were attending ANC follow up services. This finding is consistent with a study done in Gondar university hospital, Northwest Ethiopia [18]. It is possible that mothers who had ANC follow up have a better chance to get information about pregnancy preparedness and how to avert risk factors.

The odds ratio revealed that pregnant women who have suffered intimate partner violence were four times more likely to experience depression during pregnancy than those without this history. This finding is consistent with a study done in state of South Minas Gerais, Brazil [34]. This might be due to women fear of divorce, victims feeling of guilt and shame which is an aggravating factor that contributes to the occurrence of depression.

Pregnant women who had history of intake psychoactive substance were 6 times more likely to experience antenatal depression than those who were not intake psychoactive substance. This finding is consistent with a study done in Nigeria in 2016 [29]. This might be psychoactive substance like alcohol is a depressant. So, any amount of intake during pregnancy can lead to depression.

### Conclusions

More than one in six pregnant women had depression. Attending ANC during their pregnancy, conduct pregnant women husband conversion on the importance of intimate partner support and family planning services should be strengthened to avoid unplanned pregnancy. Therefore, considering antenatal depression as one component of antenatal care service and strengthen the risk prevention activities need crucial attention.

## Limitation of the study

Due to stigma towards mental illness, some participants might not actually report what they feel. It might incur social desirability bias.

## Data Availability

The data sets generated during the current study are available from corresponding author on reasonable request.

## Abbreviations

ANC: antenatal care
BDI: Beck Depression Inventory
CMD: common mental disorder
EPDS: Edinburg Postnatal Depression Scale
IPV: intimate partner violence
MCH: maternal and child health
ROC: receiver operator characteristics
SDG: Sustainable Development Goal
WHO: World Health Organization
